# Sex-Linked Chromosome Heterozygosity in Males of *Tityus confluens* (Buthidae): A Clue about the Presence of Sex Chromosomes in Scorpions

**DOI:** 10.1371/journal.pone.0164427

**Published:** 2016-10-26

**Authors:** Renzo Sebastián Adilardi, Andrés Alejandro Ojanguren-Affilastro, Liliana María Mola

**Affiliations:** 1 Laboratorio de Citogenética y Evolución, Departamento de Ecología, Genética y Evolución, IEGEBA (CONICET-UBA), Facultad de Ciencias Exactas y Naturales, Universidad de Buenos Aires, Ciudad Autónoma de Buenos Aires, Argentina; 2 División de Aracnología, Museo Argentino de Ciencias Naturales “Bernardino Rivadavia” – CONICET, Ciudad Autónoma de Buenos Aires, Argentina; Leibniz-Institute of Plant Genetics and Crop Plant Research (IPK), GERMANY

## Abstract

Scorpions of the genus *Tityus* show holokinetic chromosomes, achiasmatic male meiosis and an absence of heteromorphic sex chromosomes, like all Buthidae. In this work, we analysed the meiotic behaviour and chromosome rearrangements of a population of the scorpion *Tityus confluens*, characterising the cytotypes of males, females and embryos with different cytogenetic techniques. This revealed that all the females were structural homozygotes, while all the males were structural heterozygotes for different chromosome rearrangements. Four different cytotypes were described in males, which differed in chromosome number (2n = 5 and 2n = 6) and meiotic multivalent configurations (chains of four, five and six chromosomes). Based on a detailed mitotic and meiotic analysis, we propose a sequence of chromosome rearrangements that could give rise to each cytotype and in which fusions have played a major role. Based on the comparison of males, females and a brood of embryos, we also propose that the presence of multivalents in males and homologous pairs in females could be associated with the presence of cryptic sex chromosomes, with the male being the heterogametic sex. We propose that the ancestral karyotype of this species could have had homomorphic XY/XX (male/female) sex chromosomes and a fusion could have occurred between the Y chromosome and an autosome.

## Introduction

Buthidae is the most diverse family of scorpions of the Parvorder Buthida, which also includes the poorly diversified families Chaerilidae and Pseudochactidae, and is clearly separate from the rest of the extant scorpion families [1]. The New World buthid genus *Tityus* C. L. Koch 1836 is the most diversified of the order, with more than 200 species described. It occurs in Central and South America, and is particularly diversified in tropical areas. In the southern and more temperate parts of South America, the presence of this genus is comparatively marginal, with only a few species present in the area [2].

Cytogenetic studies in the Bothriuridae, Buthidae, Scorpionidae and Urodacidae families of scorpions revealed the presence of achiasmatic male meiosis and a lack of recognizable sex chromosomes [3–10]. The only references to sex chromosomes in scorpions were in the mitosis of *Leiurus quinquestriatus* (Ehrenberg 1828) (Buthidae) and *Nebo hierichonticus* (Simon 1872) (Scorpionidae) [11, 12], where the presence of an XY/XX system is assumed but without any evidence to support it, and in the meiosis of *Tityus bahiensis* (Perty 1833) [13].

Buthidae is the only family of scorpions with holokinetic chromosomes, i.e. chromosomes without a primary constriction. In holokinetic systems, the main chromosome rearrangements involved in karyotype evolution are fusions and fragmentations, as well as polyploidy in the monocotyledonous plant families Juncaceae and Cyperaceae [14–17]. These rearrangements would theoretically allow great chromosome number variation, since this type of chromosomes does not present the restriction that the centromere imposes (i.e. formation of dicentric and/or acentric chromosomes). A restriction that could be present in fragmentations is the requirement of telomere healing, but recent studies indicated a telomerase-based mechanism of chromosome healing in holokinetic species [18–20]. However, chromosome numbers in animal taxa are not as diverse as in monocotyledonous plants [14–16]. While reciprocal translocations have been described only in spontaneous mutants from different holokinetic taxa, polymorphisms and polytypisms for reciprocal translocations are common features in scorpions of family Buthidae [5, 8, 21]. White [22] proposed that the chances of the establishment of translocations are greater in the phylogeny of species with holokinetic chromosomes, since all the products of this type of rearrangement are transmissible at meiosis, without dicentric or acentric chromosomes. From another point of view, Darlington [23] proposed that the maintenance of interchange heterozygosity requires only inbreeding, regardless of the kinetic organization of the chromosomes.

*Tityus* is the most cytogenetically studied genus of Buthidae, with 18 species analysed. It presents a wide interspecific variation in chromosome number, from 2n = 5 to 2n = 20, and most of the species present different multivalent associations at meiosis I [7, 8, 24, 25]. Nevertheless, few studies have been performed in polymorphic populations of Buthidae presenting different types of multivalents in males in order to explain how they could have originated within each population (e.g. [5, 24]). Multivalents in scorpions are often theoretically discussed, without aiming to reconstruct the type and probable order of chromosome rearrangements involved in their origin. Another question that arises when studying polymorphic populations is whether the same chromosome rearrangements are present in males and females and how these are being transmitted across generations. There is thus a need to perform population studies that include both sexes and their offspring. The lack of a population approach in Buthidae cytogenetic studies may also have resulted in an underestimation of intrapopulation rates of chromosome rearrangements [8].

*Tityus confluens* Borelli 1899 occurs in dry Chaco environments of northern Argentina, Bolivia, southern Brazil and Paraguay [2, 26]. Its identity and distribution have been clearly established by Maury [26], who re-described the species with modern standards. *Tityus confluens* has been included in the “*confluens”* species complex [27–29], which contains several closely related species, all of which are remarkably similar. This morphological similarity, together with the difficult taxonomy of the genus, has led to some identity confusion [8, 30]. Previous studies of two male specimens identified as *T*. *confluens* from northern Brazil revealed 2n = 13, with five bivalents and a trivalent at meiosis I [8]; however, due to the locality where these specimens were collected, far from the actual distribution of *T*. *confluens* [26, 29], they most probably belong to a different species of the complex “*Tityus confluens*”.

In this work, for the first time we cytogenetically analysed a population of *T*. *confluens* from northern Argentina. We studied the karyotype of males and females and the offspring of one of these females. Four different cytotypes were described in males, which differ in chromosome number and meiotic multivalent configurations, while only one homozygous cytotype was found in females. Based on a detailed mitotic and meiotic analysis by Giemsa staining, C-banding, silver staining and FISH with 28S rDNA and (TTAGG)_*n*_ telomeric probes, we propose a sequence of chromosome rearrangements that could give rise to each cytotype. Based on the comparison of males, females and embryos, we also propose that the presence of multivalents in males and homologous pairs in females could be associated with the presence of cryptic sex chromosomes.

## Materials and Methods

Specimens of *T*. *confluens* were collected at night using UV lamps, in the “Reserva Natural Formosa” (Formosa province, Argentina) (24° 18.679'S, 61° 48.774'W); this protected area belongs to the “Administración de Parques Nacionales”, from which the necessary collection and transportation permits were obtained. The studied sample consisted of 13 males, six females and 19 embryos (of one of the females). The specimens were carried alive to the laboratory and their testes or ovariuteri were dissected in insect saline solution (154 mM NaCl, 5.63 mM KCl, 2.25 mM CaCl_2_, 2.38 mM NaHCO_3_) and then incubated in hypotonic solution (75 mM KCl) for 30 min. Tissue fixation and cytogenetic preparations were performed according to [25]. In the case of the pregnant female, each embryo was isolated from the ovariuterus after fixation and analysed separately.

Standard staining was carried out with 5% Giemsa solution in distilled water for 12–15 min. The C-banding was performed according to [31] and then stained with DAPI (4′,6-diamidino-2-phenylindole). The study of the nucleolar organiser regions (NORs) was carried out by silver-staining technique according to [32].

Fluorescence *in situ* hybridisation (FISH) was performed with 28S rDNA and (TTAGG)_*n*_ telomeric probes. Unlabelled 28S ribosomal probes were obtained by PCR using genomic DNA from *Tityus argentinus* (Borelli, 1899) as a template, as described by [25]. The telomeric probe was generated by PCR in the absence of a template [33]. The protocol was performed according to [34] with minor modifications in the design of primers and PCR mix as described by [10]. Labelling of both probes was carried out by PCR with biotin-16-dUTP. FISH was performed as described by [35] for indirect labelling and antibody detection. After overnight hybridisation, the probes were detected with streptavidin-Cy3 conjugate (Sigma, St Louis, MO, USA). The preparations were counterstained with DAPI and mounted in Vectashield (Vector, Burlingame, CA, USA). The slides were examined under a Leica DMLB microscope equipped with a Leica DFC350 FX monochrome digital camera. Pictures were pseudo-coloured and processed with Adobe Photoshop CS5. To compare the different karyotypes of males, females and embryos, chromosome measurements were made in well-spread cells at mitotic prometaphase or metaphase using ImageJ software (http://imagej.nih.gov/ij/). The cells analysed corresponded to slides subjected to FISH with rDNA probes or C-banding, in which NOR chromosomes could be unequivocally identified. The relative length of each chromosome was calculated as a percentage of total diploid complement length (%TCL).

## Results

Males of *Tityus confluens* presented intrapopulation variations of chromosome number and meiotic configuration. The study of spermatogonial mitosis and male meiotic stages allowed the characterisation of four different cytotypes, designated with letters A to D.

**Fig 1:**
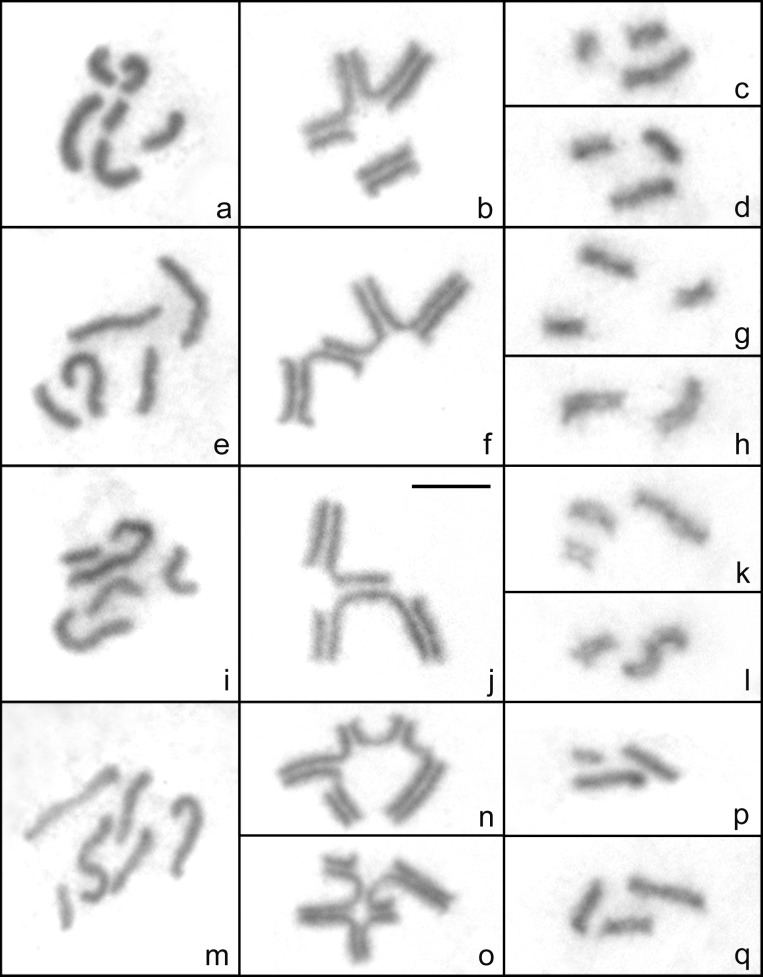
Giemsa staining of mitosis and meiosis of males of *Tityus confluens*. Cytotype A (a-d): a. Mitotic metaphase (2n = 6); b. Postpachytene (II+IV); c, d. Metaphases II (n = 3). Cytotype B (e-h): e. Mitotic prometaphase (2n = 5); f. Postpachytene (V, version 1); g, h. Metaphases II (n = 3, n = 2). Cytotype C (i-l): i. Mitotic prometaphase (2n = 5); j. Postpachytene (V, version 2); k, l. Metaphases II (n = 3, n = 2). Cytotype D (m-q): m. Mitotic prometaphase (2n = 6); n. Postpachytene (VI, chain form); o. Postpachytene (VI, ring-bearing form) p, q. Metaphases II (n = 3). Scale bar = 10μm.

**Cytotype A** was observed in eight males presenting 2n = 6 holokinetic chromosomes, with two large chromosomes of different size, three medium and one smaller medium chromosome (Fig 1a, Table 1). At postpachytene, one medium bivalent and one chain quadrivalent composed of two large, one medium and one slightly smaller medium chromosome were observed (II+IV) (Fig 1b). All metaphase II cells showed n = 3, either with the small, a medium and the largest chromosome (Fig 1c) or with two medium and the other large chromosome (Fig 1d). **Cytotype B** was found in three males and presented 2n = 5, with three large chromosomes decreasing in size, and two medium chromosomes (Fig 1e, Table 1). At postpachytene, a chain of five chromosomes was observed (V, version 1) (Fig 1f). Metaphase II cells presented n = 3 with one large and two medium chromosomes (Fig 1g) or n = 2 with the two larger chromosomes (Fig 1h). **Cytotype C** was described from one male that presented 2n = 5, with two large chromosomes of different size, two medium and one smaller medium chromosome (Fig 1i, Table 1). At postpachytene, a chain of five elements, different to that of cytotype B, was observed (V, version 2). In this version, the largest chromosome was synapsed with one medium, one small and almost half of the other large chromosome. The latter, in turn, was synapsed with the other medium chromosome, giving as a result a ramified chain (Fig 1j). Cells at the second meiotic division regularly presented n = 3, with one large, one medium and one small chromosome (Fig 1k) or n = 2 with the largest and one medium chromosome (Fig 1l). **Cytotype D** was found in one male that presented 2n = 6, with three large chromosomes decreasing in size, two medium and one smaller medium chromosome (Fig 1m, Table 1). Postpachytene cells showed one hexavalent (VI), which was observed in two different forms: one as a chain (Fig 1n) and the other as a ring with two branches (Fig 1o). The ring is produced by an interstitial synapsed region between the two larger chromosomes and involves two complete chromosomes (one large and one medium). The two larger chromosomes, in turn, partially synapse with one small and one medium chromosome, producing the two branches (Fig 1o). The chain and the ring-bearing hexavalent forms were observed in 79.5% and 20.5% of the postpachytene cells analysed (N = 151), respectively. Metaphase II cells always exhibited n = 3, either with two large and one small chromosome (Fig 1p), or with two medium and one large chromosome (Fig 1q).

**Table 1:** Chromosome measurements of males, females and embryos of *Tityus confluens*. Relative lengths expressed as percentage of total diploid complement length (%TCL). Mean values and standard deviations (SD) are given. Measurements are ordered by decreasing length and do not necessary imply homology between different cytotypes.

Males								Females		Embryos			
Cytotype A (N = 15)		Cytotype B (N = 11)		Cytotype C (N = 10)		Cytotype D (N = 10)		2n = 6 (N = 10)		2n = 6 (N = 10)		2n = 5 (N = 12)	
%TCL	SD	%TCL	SD	%TCL	SD	%TCL	SD	%TCL	SD	%TCL	SD	%TCL	SD
24.3	1.8	25.1	1.3	34.6*	1.1	22.6*	1.8	21.3	0.6	21.6	1.2	26.0	2.0
21.2	1.1	23.4*	1.9	24.7	1.6	21.3	2.0	19.4	0.8	19.7	1.2	23.2*	1.6
15.7	1.1	21.4	1.5	15.2	1.2	18.4	1.5	15.8	0.7	15.7*	1.3	20.0	1.7
14.5*	0.7	15.9	2.2	14.9*	0.9	14.6	1.7	15.1*	0.3	15.3	1.4	15.9	1.6
13.3*	0.8	14.3*	1.2	10.7	1.1	14.4*	1.2	14.3	0.5	13.9*	1.6	14.9*	1.1
11.0	0.8					8.7	1.0	14.1*	0.9	13.8	1.0		

N number of measured cells,

* NOR chromosomes

**Fig 2:**
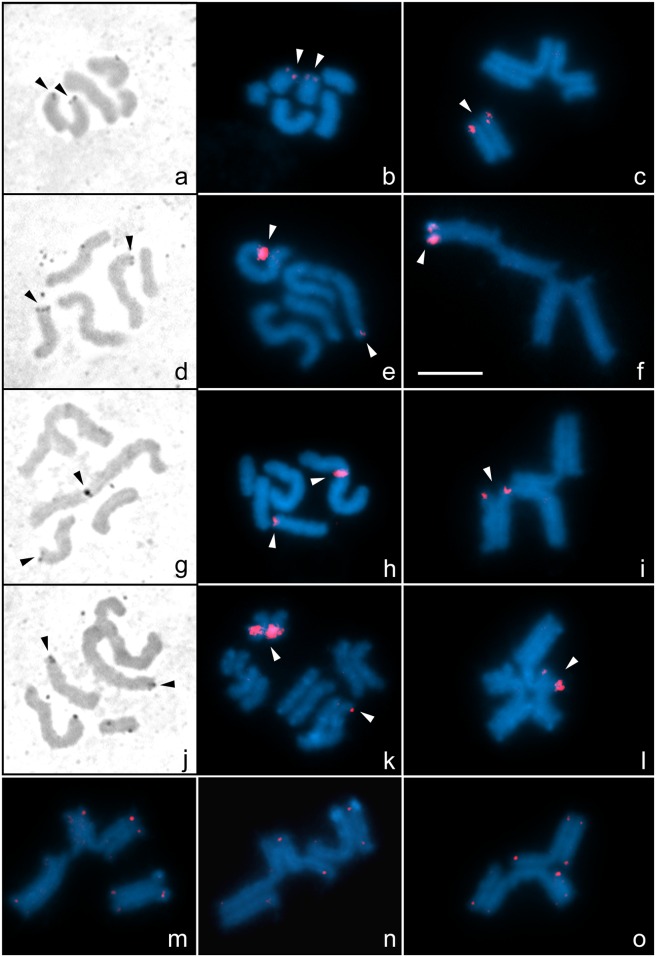
Silver staining (a, d, g, j), FISH with 28S rDNA (b, c, e, f, h, i, k, l) and (TTAGG)_*n*_ telomeric probes (m, n, o) in males of *Tityus confluens*. Cytotype A: a, b. Mitotic prometaphase (2n = 6); c, m. Postpachytene (II+IV). Cytotype B: d, e. Mitotic prometaphase (2n = 5); f, n. Postpachytene (V, version 1); Cytotype C: g, h. Mitotic prometaphase (2n = 5); i, o. Postpachytene (V, version 2). Cytotype D: j. Mitotic prometaphase (2n = 6); k. Early mitotic anaphase (2n = 6); l. Postpachytene (VI). Black arrowheads indicate Ag-NORs. White arrowheads indicate 28S rDNA hybridisation signals. Scale bar = 10 μm.

Silver staining revealed two NORs in male mitotic cells of the four cytotypes (Fig 2a, 2d, 2g and 2j). Fluorescence *in situ* hybridisation with 28S rDNA probes presented results consistent with the number and position of NORs. In **cytotype A**, rDNA clusters were detected in one terminal region of the medium pair that constitutes the bivalent (Fig 2b and 2c). In **cytotype B**, rDNA signals were detected at the terminal region of one large and one medium chromosome (Fig 2e). At postpachytene, these terminal signals were localised at one end of the pentavalent chain (Fig 2f). In **cytotype C**, one rDNA signal was observed at the terminal region of one medium chromosome, while the other was in a submedial region of the largest chromosome (Fig 2h). At postpachytene, these signals were localised in an internal region of the pentavalent chain, next to the site where the largest chromosome partially synapses with another large chromosome (Fig 2i). In **cytotype D**, the rDNA signals were in the terminal region of the largest chromosome and in one medium chromosome (Fig 2k). At postpachytene, these signals were localised in the ring in paired terminal regions of the hexavalent (Fig 2l). The signal in the largest chromosome was next to the region that occasionally synapsed with the other large chromosome in the ring-bearing form of the hexavalent (Figs 1o and 2l). In cytotype D, the signals on the large chromosome were weaker, probably indicating a lesser number of ribosomal cluster repeats (Fig 2k and 2l).

FISH with (TTAGG)_*n*_ telomeric repeats showed small signals at every terminal region of each chromosome in males of **cytotypes A**, **B** and **C** (Fig 2m–2o). Nevertheless, since the hybridisation signals were weak or undetectable in one or more terminal regions of different chromosomes of particular cells, information from different postpachytene cells had to be gathered to confirm the presence of signals at every telomeric region (S1 Fig). No interstitial telomeric signals were detected. Slides from the male with **cytotype D** lacked reliable hybridisation signals with this probe.

**Fig 3:**
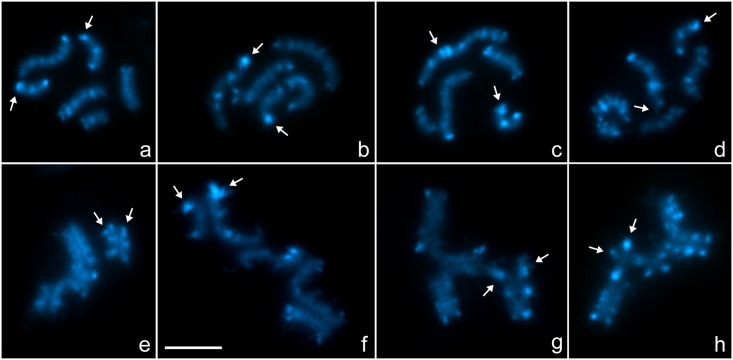
C-banding of mitotic prometaphase (a-d) and postpachytene (e-h) cells of males of *Tityus confluens*. Cytotype A: a. 2n = 6; e. II+IV. Cytotype B: b. 2n = 5; f. V version 1. Cytotype C: c. 2n = 5; g. V version 2. Cytotype D: d, 2n = 6; h. VI. White arrows indicate C-bands corresponding to rDNA sites. Scale bar = 10μm.

C-banding on male mitotic and meiotic cells revealed abundant blocks of constitutive heterochromatin of different sizes located at terminal and interstitial regions of all chromosomes (Fig 3). The distribution and sizes of almost all C-bands allowed homologous regions of the chromosomes to be recognised at the different multivalents. In **cytotype A**, the NOR pair presented a conspicuous C-band in the NOR terminal region, a medial band and another band at the other terminal region. The largest chromosome presented one terminal and one subterminal band at one end, and some scattered smaller interstitial bands. The other large chromosome presented one terminal and one subterminal band at each terminal region, and a few smaller interstitial bands. The other medium chromosome presented small bands, one at each terminal region and one subterminal, and the smaller medium chromosome presented terminal bands at each end and one submedial band (Fig 3a and 3e). In **cytotype B**, the medium NOR chromosome presented the same banding pattern as in cytotype A. The largest chromosome presented one terminal and one subterminal band at one end, and some scattered smaller interstitial bands. The large NOR chromosome presented a conspicuous terminal band, followed by an interstitial band, a medial band and smaller submedial and terminal bands. The other large chromosome presented one terminal and one subterminal band at each terminal region and a few smaller interstitial bands. The remaining medium chromosome presented small bands, one at each terminal region and one subterminal (Fig 3b and 3f). In **cytotype C**, the medium NOR chromosome presented the same pattern as above. The largest NOR chromosome presented small terminal bands, two conspicuous submedial bands and some smaller interstitial and terminal bands. The other large chromosome presented one terminal and one subterminal band at one end, and some scattered smaller interstitial bands. The other medium chromosome presented small bands, one at each terminal region and one subterminal, while the smaller medium chromosome presented terminal bands at each end and one submedial band (Fig 3c and 3g). In **cytotype D**, the medium NOR chromosome presented the same pattern as in the other cytotypes. The largest chromosome, bearing the NOR, presented terminal bands and one conspicuous submedial band followed by smaller interstitial bands. The second largest chromosome presented one terminal and one subterminal band at each terminal region and a few smaller interstitial bands. The third largest chromosome presented terminal bands and some small interstitial bands. The other medium chromosome presented small bands, one at each terminal region and one subterminal and the smaller medium chromosome presented terminal bands at each end and one submedial band (Fig 3d and 3h).

In **cytotypes A, B** and **C**, the rDNA clusters were in the same regions as the most conspicuous C-bands. In **cytotype D**, only the terminal rDNA site in the medium chromosome colocalised with a conspicuous C-band, while the rDNA site of the large chromosome colocalised with a small C-band.

**Fig 4:**
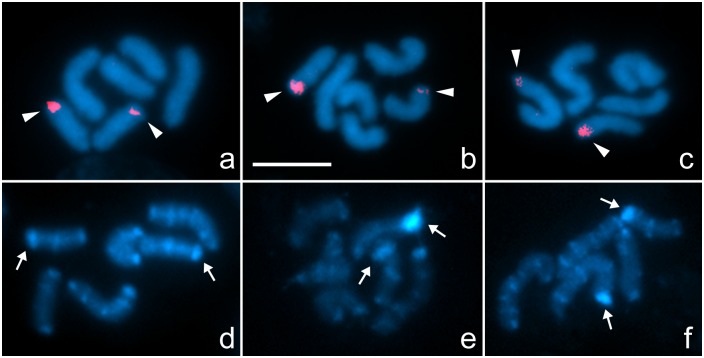
FISH with 28S rDNA probes (a-c) and C-banding (d-f) of females and embryos of *Tityus confluens*. a, d. Female mitotic prometaphase (2n = 6); b, e. Embryonic mitotic prometaphase (2n = 6); c, f. Embryonic mitotic prometaphase (2n = 5). White arrowheads indicate 28S rDNA hybridisation signals. White arrows indicate C-bands corresponding to rDNA sites. Scale bar = 10 μm

Mitotic cells from six females presented 2n = 6, with two large and four medium chromosomes (Fig 4a and 4d, Table 1). No meiotic cells were found on preparations from these females. The complete brood of 19 embryos of one of these females was cytogenetically examined. Variations in chromosome number were observed: eight embryos presented 2n = 6, with two large and four medium chromosomes (Fig 4b and 4e, Table 1), and seven embryos presented 2n = 5, with three large chromosomes decreasing in size, and two medium chromosomes (Fig 4c and 4f, Table 1). Four embryos lacked adequate mitotic cells for analysis.

FISH with 28S rDNA probes on mitotic cells from females and embryos always showed two signals. In females and embryos with 2n = 6, the rDNA signals were detected at the terminal region of two medium chromosomes (Fig 4a and 4b). In embryos with 2n = 5, the rDNA signals were detected at the terminal region of one large and one medium chromosome (Fig 4c).

C-banding of mitotic cells from females and embryos with 2n = 6 revealed a similar pattern, in which three chromosome pairs could be recognised. The medium NOR pair presented the same banding pattern as in males of cytotype A. The largest pair presented a pattern characterised by one terminal and one subterminal band at each terminal region, and a few smaller interstitial bands, similar to the largest chromosome of cytotype A. The other medium pair also presented bands, one at each terminal region and one submedial band, similar to the smaller medium chromosome of cytotype A (Fig 4d and 4e). In cells of embryos with 2n = 5, the C-banding pattern was similar to that of males of cytotype B, and the conspicuous bands also colocalised with rDNA sites (Fig 4f).

## Discussion

In this work, we analysed the meiotic behaviour and chromosome rearrangements of a population of *T*. *confluens*, characterising the cytotypes of the males, females and embryos with different cytogenetic techniques. This study allows us to postulate the origin of the different cytotypes and propose a model of cryptic sex chromosome system associated with male-linked chromosome heterozygosity.

### Multivalent segregation at meiosis I

The variation of chromosome size within multivalents, rDNA sites and constitutive heterochromatin localization allowed us to analyse the segregation of multivalents at meiosis I. The constitution of the different metaphase II cells of each male cytotype indicates a pre-reductional and balanced segregation. Thus, the fertility of these males would not be affected at this level as a consequence of mis-segregation from multivalents. Shanahan [5] proposed that the chromosomes involved in multivalents would adopt an alternate “above and below” orientation at metaphase I, ensuring a balanced segregation. The ability to undergo balanced segregation has also been described in many Buthidae species with multivalents at male meiosis [8, 10, 24], suggesting that this is a conserved feature of this group.

### Nucleolar organiser regions

Most species studied of Buthidae present one terminal ribosomal DNA cluster in one chromosome pair (or two homologous chromosome segments, in the case of rearrangements), often associated with a conspicuous heterochromatin block [8, 10, 24, 25]. The specimens of *T*. *confluens* analysed here follow this general pattern, with the exception of the male of cytotype C, which presents an interstitial NOR. Schneider et al. [24] described one male and one female of *T*. *bahiensis* (also with 2n = 5) that showed one medial NOR in a large chromosome that was twice the size of the other four chromosomes, and a subterminal NOR in another chromosome. In the male of *T*. *confluens* of cytotype C studied here, as in the two specimens of *T*. *bahiensis* mentioned above, the presence of submedial or medial NORs is explained by a fusion of the NOR end of one chromosome with another non-homologous large chromosome.

### Origin of the different cytotypes

Previously, only two studies have been performed in polymorphic populations of Buthidae scorpions with different types of multivalents to provide an explanation of how they could have originated. Shanahan [5] studied males of an isolated large population of *Lychas marmoreus* (C. L. Koch 1844) (2n = 14) from Kangaroo Island (Australia) with mainly structural homozygotes (7II) and also different heterozygotes for reciprocal translocations. Analysing only chromosome sizes, she proposed that sequential translocation events gave rise to the individuals with 4II+IV, 4II+VI and 2II+X. She also described an isolated small population of *Lychas variatus* (Thorell 1877) (2n = 14) in which all males showed one bivalent and a ring of 12 chromosomes, and postulated that the ring appeared to be fixed and established by a balanced lethal system. The females of this population showed mitotic chromosomes of the same sizes as males, which led the author to suggest that the females were also structural heterozygotes.

Schneider et al. [24] proposed that the diversity of diploid numbers (2n = 10, 9, 6 and 5) of a population of *T*. *bahiensis* from Piracicaba (Brazil) probably arose from chromosome fragmentations and/or fusions. The occurrence of these types of rearrangements was corroborated by the inverse ratio between chromosome number and size and by the different multivalents observed at male meiosis. All the mitotic cells of females that they analysed showed the same chromosome numbers and sizes as the males with 2n = 6 (and three bivalents), or with 2n = 5 (and two bivalents and a trivalent); the scant heterochromatin content hindered individual chromosome identification.

**Fig 5:**
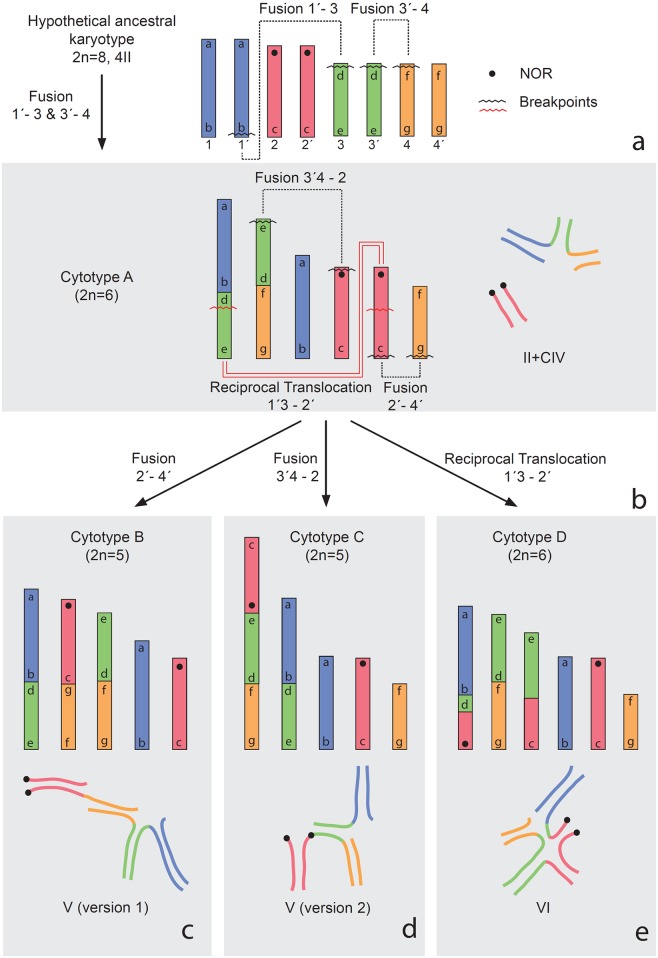
Diagram of the chromosome rearrangements that could give rise to each cytotype. a. Hypothetical ancestral karyotype and possible rearrangements that gave rise to cytotype A; b. Cytotype A and possible rearrangements that gave rise to the three other cytotypes; c. Cytotype B; d. Cytotype C; e. Cytotype D. Colours indicate the four different chromosome pairs of the hypothetical ancestral karyotype. Idiograms of cytotype A-D are in accordance with relative lengths shown in Table 1. Letters inside the chromosomes indicate terminal regions. Schematic representations of cells in b, c, d and e correspond to those shown in Fig 2c, 2f, 2i and 2l, respectively.

In the present work, considering chromosome size, C-banding pattern (in mitosis and meiosis) and NORs localization, we postulate the following origin for the different cytotypes of *T*. *confluens* described here: a hypothetical ancestral complement with four pairs of chromosomes (2n = 8), with a larger chromosome pair (pair 1), one medium pair bearing the NORs (pair 2) and two slightly smaller medium pairs of similar size (pair 3 and 4) (Fig 5a).

**Cytotype A** may have arisen from this hypothetical complement by two independent fusions. One would have involved the fusion of one large chromosome with one member of the smaller medium chromosomes (pair 3). The second would have involved the fusion of the other member of pair 3 with a chromosome of the other pair of smaller medium chromosomes (pair 4) (Fig 5a). These fusions would have created a quadrivalent chain at meiosis I, which involves the two large fused chromosomes of different size and two chromosomes of smaller and different size (i.e. the largest and a smaller medium chromosome of the hypothetical complement) (Fig 5a and 5b). The NOR pair would not be involved in this rearrangement.

The other cytotypes may have arisen independently from specimens with cytotype A. The choice of this cytotype as a starting point for the origin of cytotypes B, C and D is based on the presence of a free bivalent in cytotype A, which was considered as an ancestral trait, and the fact that a single event of chromosome rearrangement can explain the formation of each of the other three cytotypes.

**Cytotype B** may have arisen by the fusion of one NOR chromosome by the terminal region without NOR with the smaller medium non-fused chromosome (Fig 5b and 5c).

In **cytotype C**, one NOR chromosome would have fused by the terminal region with NOR with the second largest fused chromosome of the quadrivalent, giving rise to an extremely large chromosome with an interstitial NOR and partial homology with three chromosomes (Fig 5b and 5d).

In **cytotype D**, a reciprocal translocation would have occurred between one NOR chromosome and the largest fused chromosome of the quadrivalent, giving rise to a chromosome that shows partial homology with the other three chromosomes (Fig 5b and 5e). The alternative forms of the hexavalent (Fig 1n and 1o) would be a consequence of occasional pairing between the small interstitial homologous regions of the largest translocated chromosome and the homologous region of the second large fused chromosome of the quadrivalent (Fig 5e). The pairing in this small region could be under spatial constraint during the pairing of the multivalent, which would account for the lower frequency of postpachytene cells with this form (21.5%).

The cytotype of the six females and of eight of the 15 embryos is composed of three homomorphic chromosome pairs (2n = 6) (Fig 4a and 4b). For the origin of this cytotype from the ancestral karyotype, it could be proposed that the only fusion that would be present is the one between medium chromosomes (Fig 5a, fusion 3´- 4). This rearrangement would have been fixed by natural selection or genetic drift, generating a structural homozygous cytotype composed by the fused pair, the largest ancestral pair and the NOR pair. On the other hand, seven embryos presented a mitotic complement similar to cytotype B of adult males. The difference between the cytotypes of males and females as well as the variation within the brood will be discussed below.

Regarding the mechanisms of chromosome rearrangements proposed for this population, both chromosome fusion and, to a lesser extent, reciprocal translocations are present. For a better understanding of the mechanism of these fusions, we searched for the presence of interstitial telomeric sequences (ITS) by FISH, which could be indicative of this type of rearrangement. The absence of ITS in cytotypes A, B and C would indicate that the chromosome break points locate at subtelomeric regions, with the telomeric repeats of both chromosomes being lost or considerably reduced and not detectable by FISH during this process. Moreover, the weak terminal telomeric signals observed in cytotypes A-C could be evidence of a low number of telomeric repeats that could be lost in the chromosome fusions. These results match the characterisation of chromosome fusions as events that involve terminal chromosome breakages, translocation between large chromosome fragments (giving rise to a fused chromosome) and loss of small terminal products, which include the telomeres [20, 36]. However, it cannot be discarded that the low efficiency of FISH with telomeric probes could have hindered the detection of small ITS. In the aphid *Myzus persicae*, telomeric and subtelomeric sequences were lost during chromosome fusion, revealing a similar mechanism as proposed here for *T*. *confluens* [19]. In other animals with holokinetic chromosomes, ITS associated with fusions were found only in the moth *Orgyia antiqua* [37].

The study of two males from Piauí (Brazil) of “*Tityus confluens*” showed 2n = 13 and five bivalents and one trivalent at postpachytene cells. The analysis of NOR regions indicated that the chromosomes involved in the origin of the trivalents were different: in one male, the NOR-bearing chromosomes were in bivalent-like elements, but in the other male, one NOR chromosome was large and the other was a medium/small chromosome, both forming part of the trivalent [8, 38]. As previously mentioned, it is probable that there was identity confusion and the specimens from Brazil do not correspond to *T*. *confluens*, but to a closely related species. Although the specimens of both localities differ in chromosome number and meiotic configurations, the prevailing rearrangement involved is chromosomal fusion. This is in accordance with the principle of karyotype orthoselection proposed by White [22], that is, the tendency of the same type of rearrangement to occur repeatedly in different chromosomes of the same species, or in certain taxonomic groups.

### Model of cryptic sex chromosomes associated with male-linked chromosome heterozygosity

The fact that all the males of the studied population of *T*. *confluens* were structural heterozygotes and all the females were homozygotes seems to indicate a case of sex-linked chromosome heterozygosity, with the male being the heterogametic sex. This could reveal the presence of a cryptic sex chromosome pair involved in the chromosome rearrangements. We suggest that chromosome pair 1 of the hypothetical ancestral karyotype is the homomorphic sex chromosome pair: XX in females and XY in males.

**Fig 6:**
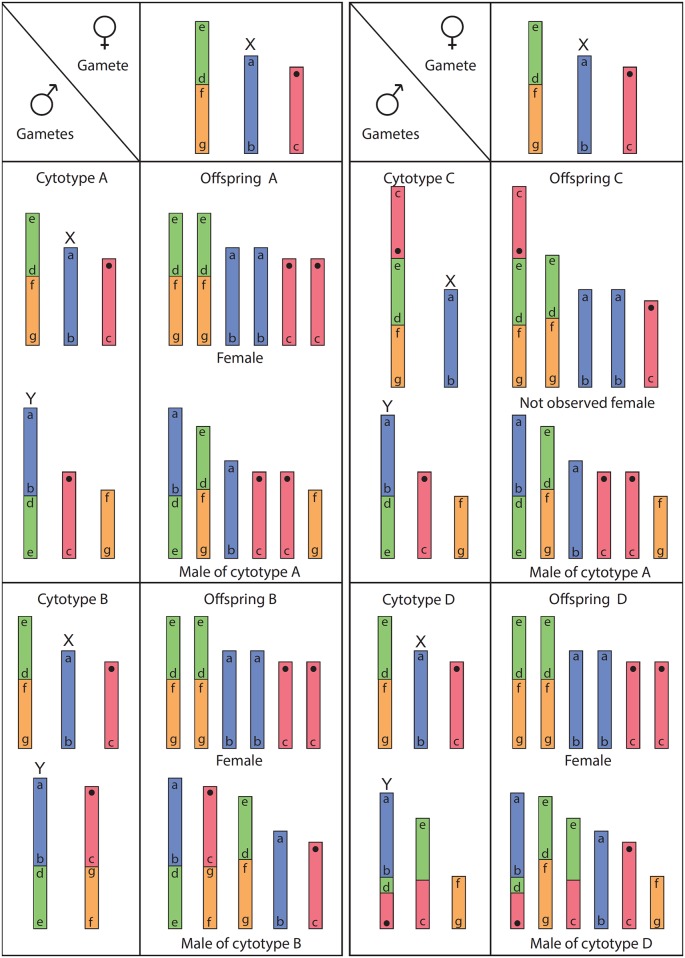
Diagram of the hypothetical offspring of the crosses between males and females of the studied population of *Tityus confluens*. Colours and sizes are in accordance with the diagram in Fig 5; the proposed X and Y chromosomes are indicated. Only alternate segregation of multivalents was considered for the formation of male gametes.

In cytotype A, the original Y chromosome could be the one involved in the fusion 1´-3 (Fig 5a) giving rise to a neo-Y chromosome, leaving the X chromosome free. Considering the chromosome fusions proposed for this cytotype (see Fig 5a and 5b), the sex chromosome system would be X_1_X_2_Y_1_Y_2_/ X_1_X_1_X_2_X_2_ (male/female), X_1_ being the original X, Y_1_ the neo-Y and X_2_ and Y_2_ (chromosomes 3´-4 and 4, respectively), both autosomes. These autosomes, due to the alternate segregation of the quadrivalent, are restricted to the gametes that determine females and males, respectively. Therefore, the segregation of the IV of this cytotype gives two types of gametes: one with X_1_, X_2_ and NOR chromosomes and the other with Y_1_, Y_2_ and the other NOR chromosome. The fusion of these gametes with the only type of female gamete (carrying X_1_, X_2_ and a NOR chromosome) will result in offspring composed of homozygous females and heterozygous males of the cytotype A (Fig 6, offspring A).

This model is reinforced by the fact that the neo-Y chromosome in cytotypes B and C remains identical to that in cytotype A. In cytotype D, only the autosomal region of the neo-Y, but not the original Y segment, participates in one reciprocal translocation. According to this model, the offspring of the cross between males from cytotypes B and D with homozygous females would give rise to structural heterozygous males with the same complement of the male progenitors, and homozygous females with the same complement of the female progenitor (Fig 6, offspring B and D). In fact, the brood of embryos of this population analysed, with a nearly equal number of specimens with 2n = 6 and female cytotype and with 2n = 5 and cytotype B (Fig 4b and 4c), support this model and represent the proposed offspring of the cross between a male of cytotype B and a homozygous female (Fig 6, offspring B).

According to this model, the offspring of the cross between males of cytotype C and homozygous females would be males of cytotype A and structural heterozygous females, which were not observed in the population (Fig 6, offspring C). At meiosis, these females would present the sex bivalent and an autosomic trivalent. The fact that no females with this karyotype were observed could be due to their low frequency: considering the sample analysed, the proportion of males that would generate these unobserved females is 1/13, while the remaining 12 males would generate homozygous females. Another explanation could be that this type of females is less viable due to a non-adaptive gene combination associated with the autosomal multivalent.

The model proposed here may be supported by results obtained by Mattos et al. [8, 38] in a population of *T*. *bahiensis* from Paraná (Brazil), in which all the individuals presented 2n = 6, but male and female karyotypes differed. Seven males presented five large chromosomes (one of them larger) and one remarkably small chromosome which formed one hexavalent at meiosis I, while five females presented six chromosomes of similar size. NORs were detected in two large chromosomes of different size in males and in two large chromosomes of similar size in females. This population could bear cryptic sex chromosomes that gave rise to structural heterozygous males and homozygous females.

There is only one meiotic study in which inferences about the existence of sex chromosomes in scorpions are made. Piza [13] described one population of *T*. *bahiensis* in which males presented 2n = 9 (II+VII) and females 2n = 10 and proposed that females produced oocytes with 5 chromosomes and males were heterogametic and produced spermatozoa with 4 and 5 chromosomes. He thus proposes an incipient sex determination mechanism conditioned by a particular chromosome: the male would present only one chromosome of this type, while the female would have two. Furthermore, the author stresses that this sex chromosome is involved in the heptavalent. White [22], based on Piza’s works, also considers that the presence of multivalents with an odd number of chromosomes in scorpion males could be “somehow involved in sex determination”, where the male would be the heterogametic sex.

In this work, we also propose that the male is the heterogametic sex, but with an XY sex chromosome system in which the Y chromosome could be fused with an autosome, and thus involved in multivalent associations, with even or odd numbers of chromosomes. The results analysed by Piza [13] could be reinterpreted according to our model, considering that the Y chromosome is involved in the fusions and the sex chromosome system is XY/XX (male/female) instead of X0/XX (male/female), as is apparent from Piza’s conclusions.

The model introduced here suggests, for the first time in the order Scorpiones, the existence of an XY/XX (male/female) sex chromosome system, with homomorphic cryptic sex chromosomes.

Increasing information on the genomes of Buthidae, as seen in the genome sequence of *Mesobuthus martensii* (Karsch 1879) [39], may provide powerful tools to study sex chromosomes. Further comparative studies on male and female genomes could help to detect sex-linked sequences and enable this data to be integrated with cytogenetic evidence. Similar approaches permitted the identification of Y chromosome genes or sex chromosomes in other groups of arthropods [40, 41]. Due to its phylogenetic position in the ancient order Scorpiones, Buthidae is of particular evolutionary interest for exploring sex determination systems in the order.

## Supporting Information

S1 Fig FISH with (TTAGG)_*n*_ telomeric probes in males of *Tityus confluens*. Additional postpachytene cells with signals at telomeric regions that presented weak or unobservable hybridisation signals at Fig 2m–2o. a. Cytotype A (II+IV); b. Cytotype B (V, version 1); c. Cytotype C (V, version 2). Scale bar = 10 μm
(TIF)
