# Supplementary figures and images for: Sex-Linked Chromosome Heterozygosity in Males of Tityus confluens (Buthidae): A Clue about the Presence of Sex Chromosomes in Scorpions
Source: PLoS One. 2016 Oct 26;11(10):e0164427. doi: 10.1371/journal.pone.0164427 (PMC5081195; doi:10.1371/journal.pone.0164427)

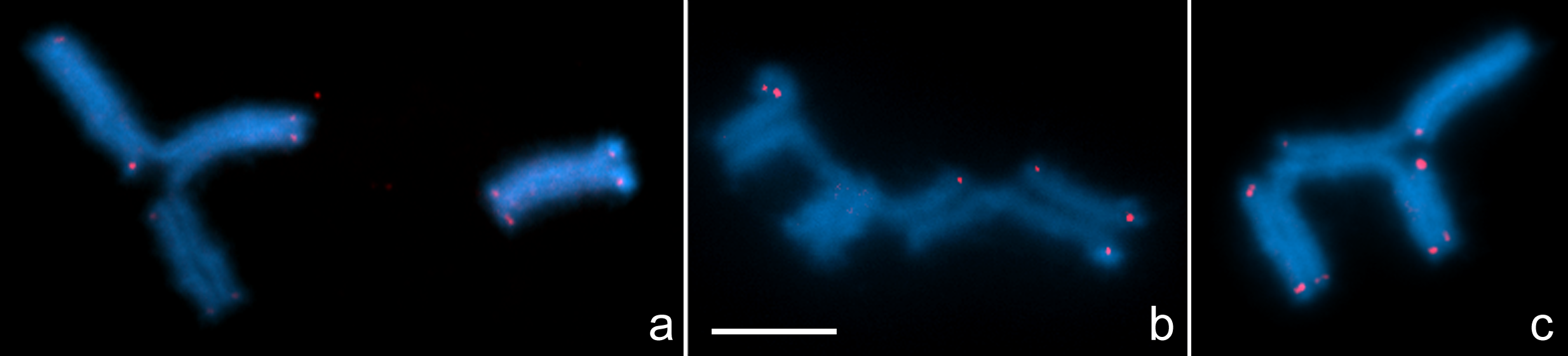

Supplement: S1 Fig — Additional postpachytene cells with signals at telomeric regions that presented weak or unobservable hybridisation signals at Fig 2m–2o. a. Cytotype A (II+IV); b. Cytotype B (V, version 1); c. Cytotype C (V, version 2). Scale bar = 10 μm (TIF) [file pone.0164427.s001.tif]
